# Cancer systems biology of TCGA SKCM: Efficient detection of genomic drivers in melanoma

**DOI:** 10.1038/srep07857

**Published:** 2015-01-20

**Authors:** Jian Guan, Rohit Gupta, Fabian V. Filipp

**Affiliations:** 1 Systems Biology and Cancer Metabolism, Program for Quantitative Systems Biology, University of California Merced, Merced, CA 95343, USA

## Abstract

We characterized the mutational landscape of human skin cutaneous melanoma (SKCM) using data obtained from The Cancer Genome Atlas (TCGA) project. We analyzed next-generation sequencing data of somatic copy number alterations and somatic mutations in 303 metastatic melanomas. We were able to confirm preeminent drivers of melanoma as well as identify new melanoma genes. The TCGA SKCM study confirmed a dominance of somatic BRAF mutations in 50% of patients. The mutational burden of melanoma patients is an order of magnitude higher than of other TCGA cohorts. A multi-step filter enriched somatic mutations while accounting for recurrence, conservation, and basal rate. Thus, this filter can serve as a paradigm for analysis of genome-wide next-generation sequencing data of large cohorts with a high mutational burden. Analysis of TCGA melanoma data using such a multi-step filter discovered novel and statistically significant potential melanoma driver genes. In the context of the Pan-Cancer study we report a detailed analysis of the mutational landscape of BRAF and other drivers across cancer tissues. Integrated analysis of somatic mutations, somatic copy number alterations, low pass copy numbers, and gene expression of the melanogenesis pathway shows coordination of proliferative events by Gs-protein and cyclin signaling at a systems level.

The Cancer Genome Atlas project aims at the comprehensive elucidation of genomic changes contributing to malignancies. The application of next-generation sequencing through whole-genome, whole-exome, and whole-transcriptome approaches revolutionized the resolution of cancer genome alterations, including nucleotide substitutions, small insertions and deletions, copy number alterations, chromosomal rearrangements, splice variants, regulation of gene expression, and viral or microbial interactions^1^. For melanoma patients, next-generation sequencing has already brought tangible advances. Identification of activating point-mutations in BRAF kinase (B-Raf proto-oncogene, serine/threonine kinase, Gene ID: 673) has now established a personalized medicine option with kinase inhibitors of mutated BRAF^2^,^3^,^4^,^5^. However, melanoma patients frequently develop resistance to BRAF inhibition^6^. In addition, melanoma subtypes with non-mutated or non-amplified BRAF, NRAS (neuroblastoma RAS viral (v-ras) oncogene homolog, Gene ID: 4893), KIT (v-kit Hardy-Zuckerman 4 feline sarcoma viral oncogene homolog, Gene ID: 3815), or MAP2Ks (mitogen-activated protein kinase kinase, Gene IDs: 5604-5609) lack molecular targets and present a need to deepen our knowledge of the molecular signature of melanoma.

Here, we describe the genomic landscape of skin cutaneous melanoma (SKCM) based on genome-wide sequencing data from 303 TCGA malignant melanoma patients. We account for the high UV-induced basal mutation rate in skin cancers and identify genes with significantly perturbed signatures. By employing a multi-step filter we suggest a modular protocol to efficiently enrich genomic drivers in melanoma. Moreover, we characterize several novel variants of known oncogenes like BRAF and relate molecular features of new potential drivers of melanoma to recurring features observed in other cancer tissues. The comprehensive analysis provides a foundation for future functional and clinical assessment of susceptibility variants in melanoma.

## Results

### Patient cohort

The TCGA SKCM cohort is focused on metastatic cases (11.6% regional skin cutaneous or subcutaneous metastatic tissue, 56.4% regional metastatic lymph node, 25.1% distant or unspecified metastatic tissue) because melanoma is most often discovered after it has metastasized. We utilized files from 299 single nucleotide polymorphism (SNP) arrays, 102 whole-genome sequencing (WGS), and 276 whole-exome sequencing (WES) datasets with normal reference samples from 303 TCGA patients between 15–90 years of age (Supplementary table 1).

### SCNA

Somatic copy number alterations (SCNAs) were analyzed using both SNP arrays and segmented low coverage whole-genome sequencing data; coverage of next-generation sequencing data sufficient for variant detection was set to 14 read-depth in metastases and 8 read-depth in normal blood-derived reference samples. All calls of cytobands by whole-genome sequencing were compared to level 3 segmented data at the TCGA data portal and confirmed by SNP array data (Supplementary tables 2–5). Overall, individual SNP arrays produced far fewer copy number calls compared to low coverage whole-genome sequencing experiments. Since low coverage whole-genome sequencing data produces more frequent calls, the number of significant SCNAs by whole-genome sequencing was lower than by SNP arrays.

**Figure 1:**
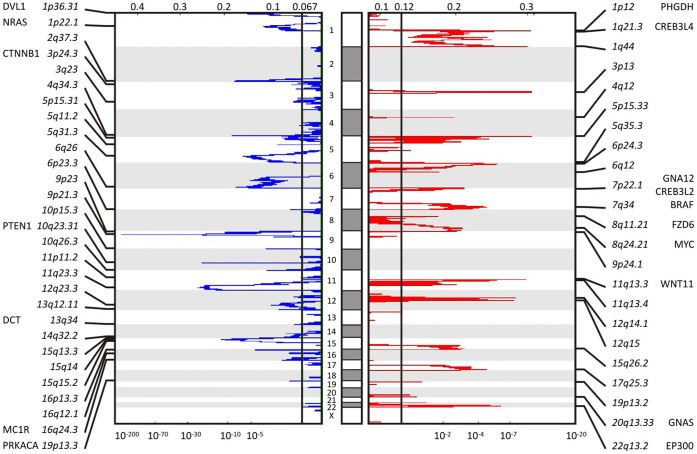
Somatic copy number alteration profiling of TCGA SKCM data shows significant focal deletions and amplifications. Somatic copy number alteration analysis identifies genomic regions that are significantly gained or lost across a set of tumors. GISTIC 2.0.21 found 21 significant arm-level results, 23 significant focal amplifications, and 29 significant focal deletions in segmented SNP array data of 292 SKCM metastatic tumor samples. Among those results, amplification of BRAF, as well as reduction of NRAS and PTEN is found. The genomic position is indicated by chromosome number in the middle panel; chromosome bands and altered genes are labeled at the sides. Normalized amplifications and deletions are labeled on top and shown in red and blue, respectively.

The tool GISTIC, genomic identification of significant targets in cancer^7^,^8^, identified 3 amplifications and 3 deletions concordantly by both SNP arrays and whole-genome sequencing; it identified 14 amplified and 13 deleted recurrent focal SCNAs detected by SNP arrays affecting 745 amplifications and 1224 deletions of genes with q-values (minimum false discovery rate at which the test may be called significant) below a threshold of 0.01 in 299 patients (Figure 1, Supplementary tables 2–5). There was significant arm-level amplification of chromosome bands 1q, 6p, 7p, 7q, 20p, and 20q detected as well as deletion of 6q, 9p, 9q, 10p, 10q, 11p, 11q, 14q, 17p with q-values below 0.01 by both SNP arrays and whole-genome sequencing. The SCNA data also revealed significant amplification of 39 miRNAs and deletion of 73 miRNAs with a gene-wise q-value below 0.01. Genes involved in pathways of mitogen-activated protein kinase (MAPK) signaling, melanogenesis, beta catenin/wingless-type (WNT), and Aurora kinase signaling are significantly deregulated, each with pathway alterations of more than 5% of the cohort size.

The most common amplification event occurs at chromosome 3 band p13, chr3:69742187-70115687, and contains MITF (microphthalmia-associated transcription factor, Gene ID: 4286) (Figure 1, Supplementary table 2–3). Focal amplification at chromosome 1 band p12, chr1:119726941-150032773, includes PHGDH (phosphoglycerate dehydrogenase, Gene ID: 26227), HMGCS2 (3-hydroxy-3-methylglutaryl-CoA synthase 2, Gene ID: 3158), NOTCH2 (notch 2, Gene ID: 4853), PDE4DIP (phosphodiesterase 4D interacting protein, Gene ID: 9659), BCL9 (B-cell CLL/lymphoma 9, Gene ID: 607). At chromosome 1 band 22q13.2, chr22:41468899-41849552, EP300 (E1A binding protein p300, Gene ID: 2033), MKL1 (megakaryoblastic leukemia 1, Gene ID: 57591), ACO2 (aconitase 2, Gene ID: 50), and RANGAP1 (Ran GTPase activating protein 1, Gene ID: 5905) are among the significantly amplified genes in melanoma with a q-value of 1.0892e-06. BRAF, EZH2 (enhancer of zeste 2 polycomb repressive complex 2 subunit, Gene ID: 2146), CREB3L2 (cAMP responsive element binding protein 3-like 2, Gene ID: 64764) at band 7q34 chr7:135929407-143664054 are amplified with a q-value of 2.2567e-05. Additional focal SCNA amplifications detected by the SNP arrays are PRKAR1B (protein kinase, cAMP-dependent, regulatory, type I, beta, Gene ID: 5575), GNA12 (guanine nucleotide binding protein (G protein) alpha 12, Gene ID: 2768), RAC1 (rho family, small GTP binding protein ras-related C3 botulinum toxin substrate 1, Gene ID: 5879) at 7p22.1, FZD6 (frizzled class receptor 6, Gene ID: 8323), MYC (v-myc avian myelocytomatosis viral oncogene homolog, Gene ID: 4609) at band 8q24.21, CCND1 (cyclin D1, Gene ID: 595), WNT11 (wingless-type MMTV integration site family, member 11, Gene ID: 7481), at band 11q13.4, GNAS (guanine nucleotide binding protein, alpha stimulating, GNAS complex locus, Gene ID: 2778), AURKA (aurora kinase A, Gene ID: 6790) at band 20q13.33, and CDK4 (cyclin-dependent kinase 4, Gene ID: 1019), MDM2 (MDM2 proto-oncogene, E3 ubiquitin protein ligase, Gene ID: 4193), RAP1B (RAP1B, member of RAS oncogene family, Gene ID: 5908) at bands 12q14.1 and 12q15 and others.

The region around chromosome 9 band p21.3, chr9:21946194-21977643, includes CDKN2A (cyclin-dependent kinase inhibitor 2A, Gene ID: 1029) and shows a significant deletion with a q-value of 4.9316e-169 (Supplementary table 4–5). Other detected gene deletions include NRAS, PRKACB (protein kinase, cAMP-dependent, catalytic, beta, Gene ID: 5567), BCL10 (B-cell CLL/lymphoma 10, Gene ID: 8915), TRIM33 (tripartite motif containing 33, Gene ID: 51592) and RBM15 (RNA binding motif protein 15, Gene ID: 64783) at band 1p22, DVL1 (dishevelled segment polarity protein 1, Gene ID: 1855), RPL22 (ribosomal protein L22, Gene ID: 6146), TNFRSF14 (tumor necrosis factor receptor superfamily, member 14, Gene ID: 8764), PRDM16 (PR domain containing 16, Gene ID: 63976) at band 1p36, CTNNB1 (catenin, cadherin-associated protein, beta 1, Gene ID: 1499), RAF1 (Raf-1 proto-oncogene, serine/threonine kinase, Gene ID: 5894) at band 3p24.3, PRKAB1 (protein kinase, AMP-activated, beta 1, Gene ID: 5564) at band 12q23.3, BRCA2 (breast cancer 2, early onset, Gene ID: 675) at band 13q12.11, RB1 (retinoblastoma 1, Gene ID: 5925), DCT (dopachrome tautomerase, Gene ID: 1638) at band 13q34, AKT1 (v-akt murine thymoma viral oncogene homolog 1, Gene ID: 207) at band 14q32.3, and MC1R (melanocortin 1 receptor, alpha melanocyte stimulating hormone receptor, Gene ID: 4157) at band 16q24. The loss of chromosome 10 band q23 containing PTEN (phosphatase and tensin homolog, Gene ID: 5728) is associated with patient age (p-value of 8.81e-05).

### Somatic mutations

The SKCM dataset comprises, after pre-processing of 276 patients (Supplementary table 1), a number of 55,462,639 incidences listed by MuTect, an algorithm for sensitive detection of mutations, of which 890,914 were identified as KEEP mutations. These KEEP mutations build the foundation for the gene-wise perturbation models. Exonic regions account for 220,031 mutations and classify as 60.1% missense, 3.8% nonsense, 0.5% frameshift, 0.1% in-frame insertion or deletions, 2.6% splice site, and 32.9% silent mutations. The SKCM dataset with a mutation rate of 18 mutations per mega base pairs (Mbp) is about 10-fold richer than other TCGA tissues (e.g. glioblastoma multiforme (GBM) has a rate of 1.8 mutations per Mbp in TCGA). Careful identification of non-synonymous mutations in combination with consideration of passenger events and basal mutation rate took the frequency of UV-associated gene mutations into account.

In order to identify potential melanoma driver genes, we established a multi-step filter for somatic mutations (Table 1, Methods). Steps included i) cohort selection at TCGA data portal, ii) mutation call of whole-exome sequencing data against their somatic references, iii) identification of recurrent and conserved positions, and iv) enrichment of mutations above the basal mutation rate using a permutation model^9^. This first set of filters ensures statistically significant enrichment of potential driver genes. To explore the biological impact of mutations we added a second set of filters to identify cancer drivers. Steps included v) relative frequencies of nucleotide mutations^10^, vi) functional mutation burden using structural modeling, vii) pathway enrichment and mutual exclusivity to known cancer drivers^11^, and viii) significance in multiple TCGA tissues.

**Figure 2:**
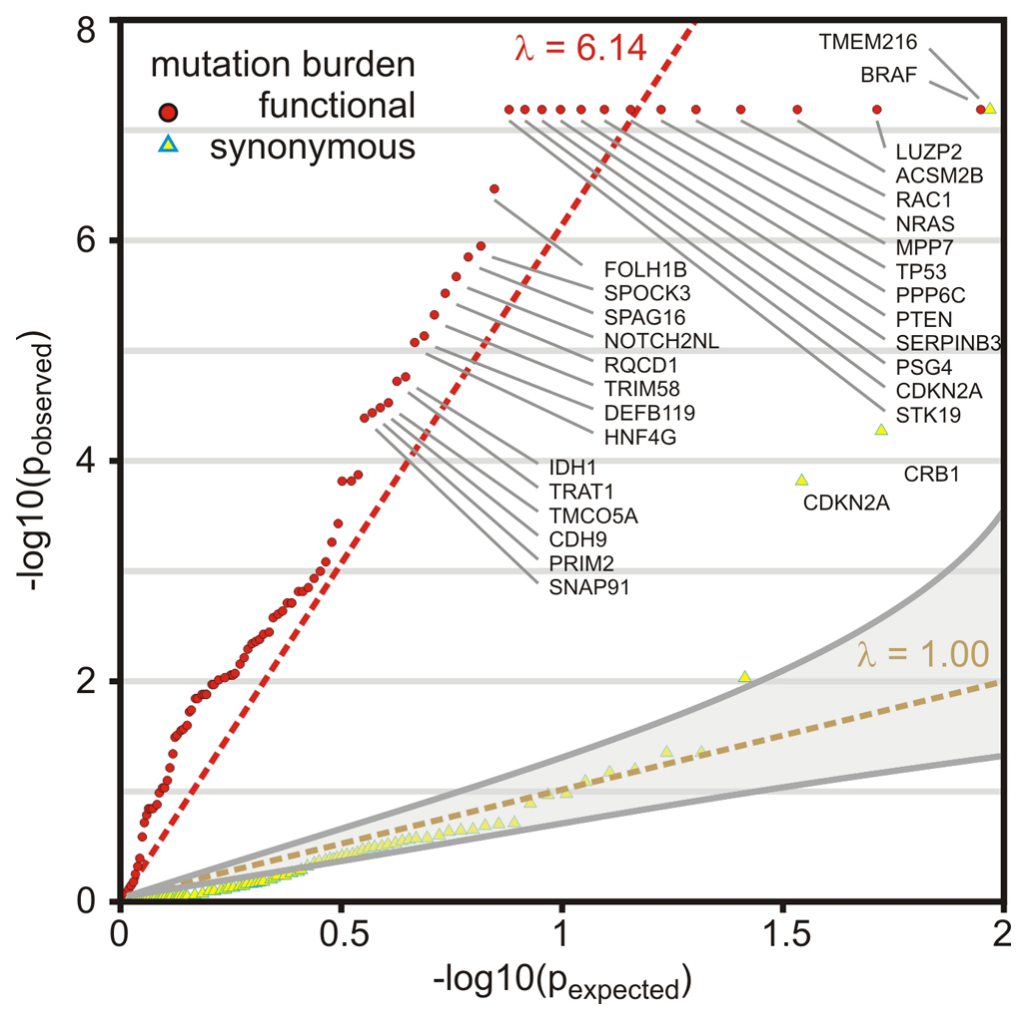
Filtered genes show significant enrichment of somatic mutations above background mutation rate. QQ-plot of mutational significance analysis is based on a permutation analysis of the background mutation rate. q-values (q = -log_10_(p)) above the diagonal indicate enrichment of somatic mutation. The diagonal y = x serves as reference where observed and expected mutational burden coincide. The significantly enriched functional mutation burden of genes passed an q-value cut-off < = 0.2 is shown as red circles. The synonymous mutation burden is shown as yellow triangles. Genes with significantly enriched synonymous mutation burden passed an q-value cut-off < = 0.2 are highlighted with blue frame. Best-fit is shown as dashed-red line (λ = 6.14) and y = x as dashed-yellow line. Gray-shaded area represents 95% confidence interval for expected p-values.

**Figure 3:**
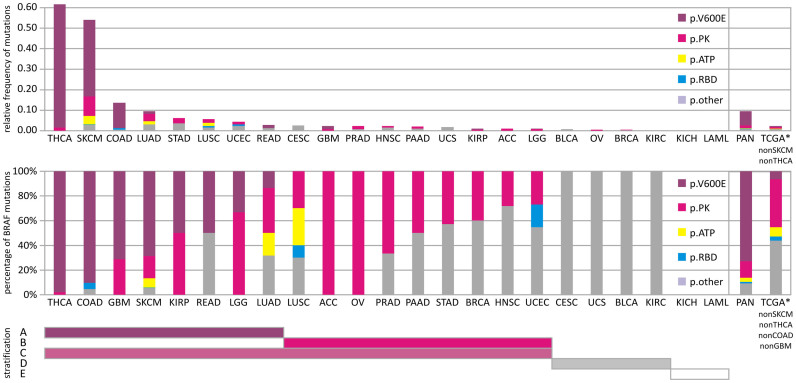
Distribution of somatic mutations and mutation types in BRAF gene across The Cancer Genome Atlas Pan-Cancer analysis project. Top panel shows the relative frequency of non-silent somatic mutations (number of observed type of somatic mutation/cohort size) of detected mutations across different human cancer tissues within TCGA. Bottom panel shows fraction of mutations sorted by affected protein domains of BRAF. The analysis includes all non-silent (protein coding missense, indels, frame-shift, stop, splice site) mutations and distinguishes mutations of V600E in purple, ATP binding site in yellow, all mutations in the protein kinase (PK) domain of BRAF but not V600E or ATP binding site in pink, RAS-binding domain (RBD) in blue, and remaining protein sequence (other) in grey. The PAN-cancer analysis covers cancer tissues: adrenocortical carcinoma (ACC), bladder urothelial carcinoma (BLCA), breast invasive carcinoma (BRCA), cervical squamous cell carcinoma and endocervical adenocarcinoma (CESC), colon adenocarcinoma (COAD), glioblastoma multiforme (GBM), head and neck squamous cell carcinoma (HNSC), kidney chromophobe (KICH), kidney renal clear cell carcinoma (KIRC), kidney renal papillary cell carcinoma (KIRP), acute myeloid leukemia (LAML), brain lower grade glioma (LGG), liver hepatocellular carcinoma (LIHC), lung adenocarcinoma (LUAD), lung squamous cell carcinoma (LUSC), ovarian serous cystadenocarcinoma (OV), pancreatic adenocarcinoma (PAAD), prostate adenocarcinoma (PRAD), rectum adenocarcinoma (READ), skin cutaneous melanoma (SKCM), stomach adenocarcinoma (STAD), thyroid adenocarcinoma (THCA), uterine corpus endometrioid carcinoma (UCEC), and uterine carcinosarcoma (UCS). Right box within panels includes analysis for PAN-cancer cohort and sub-cohort that excludes BRAF-rich cancers of more than 0.5 relative frequency, like SKCM and THCA, or of more than 50% V600E BRAF, like THCA, COAD, SKCM, GBM (TCGA*). Patient stratification: Bars below the panels mark stratification strategy of human cancers based on their BRAF genotype. A BRAF-V600E mutation; B BRAF mutation in protein kinase domain other than V600E; C BRAF-mutation in protein kinase domain; D BRAF-mutation other than protein kinase or RBD; E no BRAF mutation.

**Figure 4:**
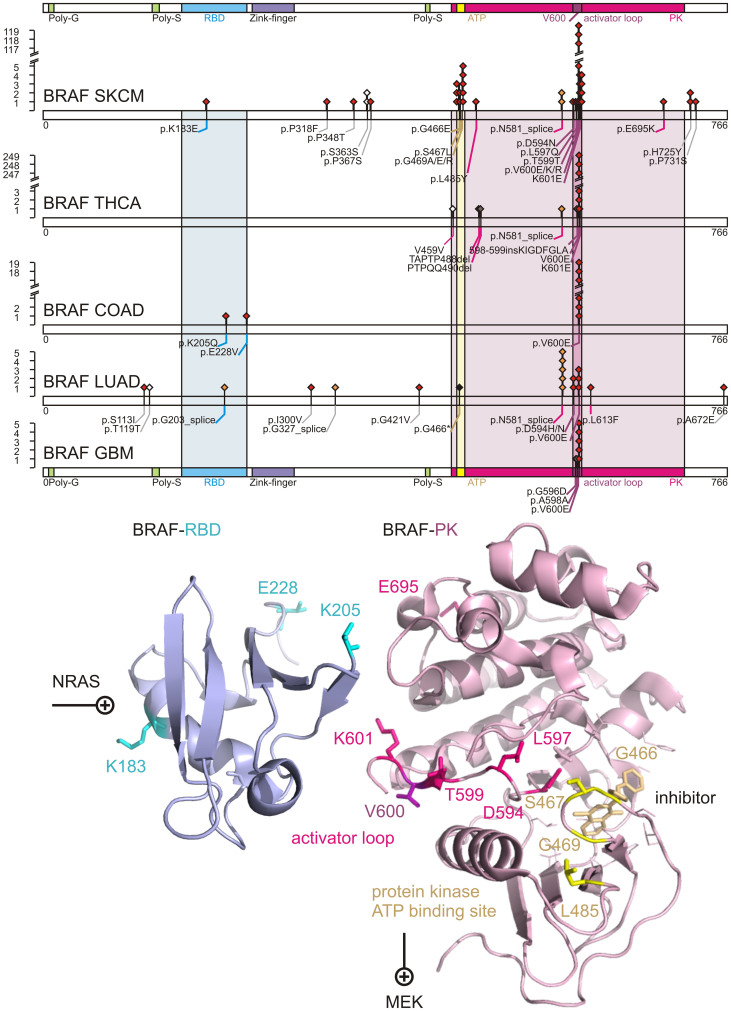
Comprehensive somatic mutational landscape of BRAF as PAN-cancer driver. Distribution of somatic mutations are shown, for five TCGA tissues with significant BRAF mutations: skin cutaneous melanoma (SKCM), thyroid adenocarcinoma (THCA), colon adenocarcinoma (COAD), lung adenocarcinoma (LUAD), glioblastoma multiforme (GBM). Diamonds indicate mutation type of non-synonymous mutations in red, splice-site mutations in orange, indels in brown, stop in black, and silent protein-coding mutations in white. Numbers refer to codons. Each filled circle represents an individual mutated tumour sample. The RAS binding domain (RBD) of BRAF is colored in magenta. The protein kinase (PK) domain of BRAF is colored in blue with the ATP binding site highlighted in yellow, and the activator loop around residue 600 in purple. Domains are colored accordingly. Residues affected by coding somatic mutations of BRAF, NCBI Gene ID 673, are depicted in sticks onto ribbon structure of 3ny5.pdb and 4e26.pdb.

**Figure 5:**
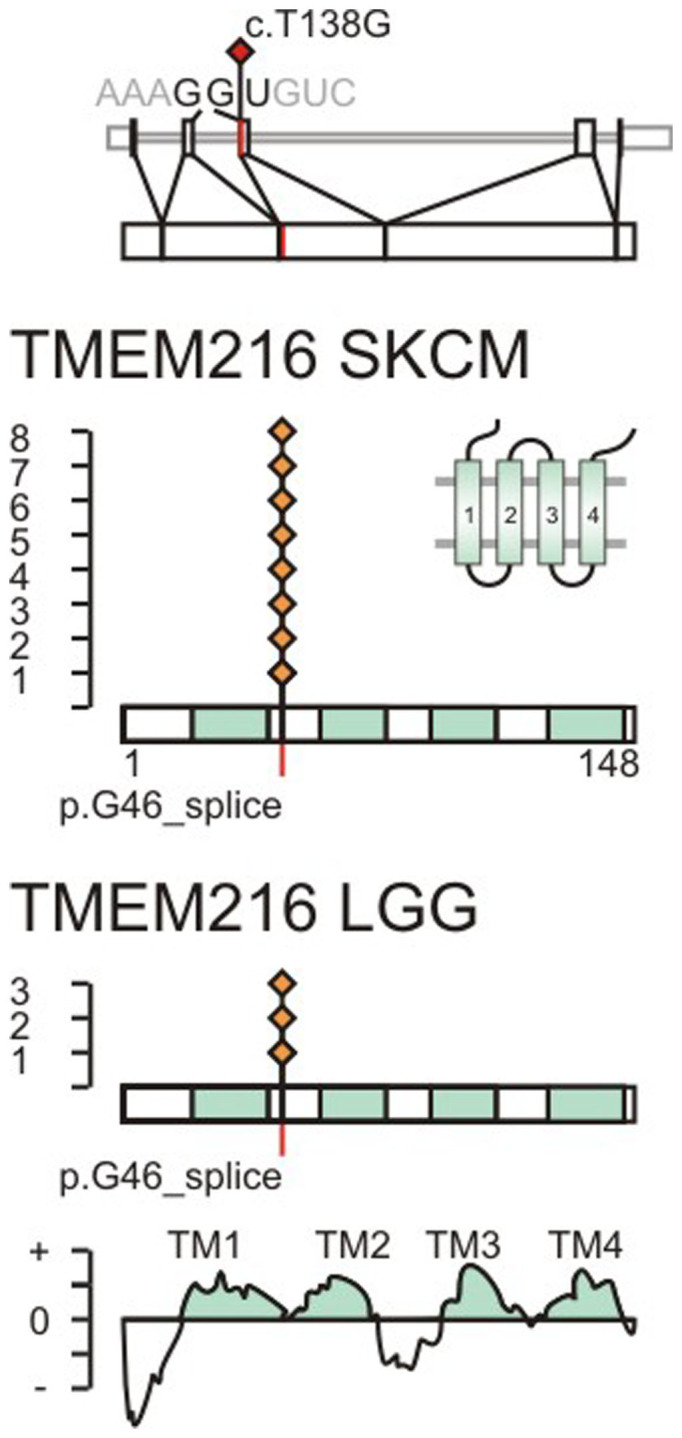
The c.T138G somatic mutation is recurring and affects the splice site of TMEM216. Splice site mutations are indicated in red on the transcript and protein sequence of TMEM216, NCBI Gene ID 51259. The somatic mutation c.T138G is located on exon 3 at the second splice site. The splice site codon 46 translates to glycine 46. Observed somatic mutations in TCGA SKCM and LGG datasets are marked by red diamonds. The position of transmembrane helices is indicated in cyan and determined based on protein family PF09799, positive segments in TMPred, and uniprot entry Q9P0N5.

In the SKCM dataset of metastatic samples with normal references, the multi-step filter analysis produced a list of 23 significantly mutated genes (22 nonsense and 1 synonymous mutation) with a q-value below 1.0e-04 (Figure 2). The multi-step filter analysis confirmed known cancer driver genes BRAF (Figure 3–4, Supplementary table 6), RAC1, NRAS, TP53 (tumor protein p53, Gene ID: 7157), CDKN2A (results in p16INK transcript), STK19 (serine/threonine kinase 19, Gene ID: 8859), PPP6C (protein phosphatase 6, catalytic subunit, Gene ID: 5537), PTEN, IDH1 (isocitrate dehydrogenase 1, Gene ID: 3417), NMS (neuromedin S, Gene ID: 129521), CDK4, and VEGFC (vascular endothelial growth factor C, Gene ID: 7424) with significantly enriched functional mutations that passed a q-value cut-off below 0.01. In addition, TMEM216 (transmembrane protein 216, Gene ID: 51259) (Figure 5, Supplementary table 7), CRB1 (crumbs family member 1, photoreceptor morphogenesis associated, Gene ID: 23418), and CDKN2A were significantly enriched genes with synonymous mutations below a q-value cut-off below 0.01. The study also highlighted genes that had never been associated with melanoma like LUZP2 (leucine zipper protein 2, Gene ID: 338645) (Supplementary information 1, Supplementary table 8), PSG4 (pregnancy specific beta-1-glycoprotein 4, Gene ID: 5672), SERPINB3 (serpin peptidase inhibitor, clade B (ovalbumin), member 3, Gene ID: 6317), SPOCK3 (sparc/osteonectin, cwcv and kazal-like domains proteoglycan (testican) 3, Gene ID: 50859), FOLH1B (folate hydrolase 1B, Gene ID: 219595), SPAG16 (sperm associated antigen 16, Gene ID: 79582), NOTCH2NL (notch 2 N-terminal like, Gene ID: 388677), TRIM58 (tripartite motif containing 58, Gene ID: 25893), RQCD1 (RCD1 required for cell differentiation1 homolog, Gene ID: 9125), and ACSM2B (acyl-CoA synthetase medium-chain family member 2B, Gene ID: 348158) below a q-value cut-off of 1.0e-04 (Supplementary tables 9–10).

### BRAF dominates the mutational landscape of melanoma

Given the predominance of BRAF mutations in metastatic melanoma^2^,^3^,^4^,^5^, we characterized the somatic mutation landscape of the BRAF gene in the SKCM dataset as well as in other TCGA cancer tissues.

The metastatic SKCM cohort of 276 patients with somatic controls contained 140 patients with non-silent mutations of BRAF and included 151 amino acid replacements affecting 18 unique residues (50% patient mutation frequency, p-value <1.00e-15, q-value <2.26e-12). The single most abundant protein-coding amino acid replacement observed in 119 of 276 samples is p.V600E, switching BRAF into a constitutively active protein kinase^2^. Besides V600E there are additional non-silent polar replacements in the activator loop (p.D594N, p.L597Q, p.V600K, p.V600R, and p.K601E). Next, we investigated whether such unprecedented diversity of BRAF mutations is specific to melanoma or common to other cancers. By calculating the relative frequency of mutations corrected for the cohort size, other BRAF-driven cancers were identified (Figure 3, Supplementary table 6). Thyroid cancer (THCA) stood out for containing frequent and recurrent somatic mutations of BRAF p.V600E with 249 of 350 cases (Figure 3, Supplementary table 6). In contrast, other significantly enriched datasets with BRAF mutations like colon adenocarcinoma (COAD), lung adenocarcinoma (LUAD), or SKCM showed mutations up to 37% in other conserved sections of the protein like the RAS-binding domain (RBD) (p.K183E, p.K205Q, p.E228V), the glycine-rich ATP binding site (p.G466E, p.S467L, p.G469A, p.G469E, p.G469R), or the protein surface connecting RBD and protein kinase (p.E695K) (Figure 4).

### Functional analysis of somatic mutations of melanoma genes

In order to identify potential melanoma drivers, we assessed functional relevance of significant somatic mutations from nucleotide signature, structure activity relationship, mutual exclusivity to known cancer drivers, and recurrence in other cancer tissues. In the context of this study aimed at characterizing the genomic landscape of melanoma, there is space to discuss somatic mutations of two new, highly significant genes, TMEM216 (Figure 5) and LUZP2 (Supplementary information 1). Both genes display q-values below 1.0e-6 after the first four steps of the mutational analysis. Their signature of nucleotide replacement related to UV radiation deviated from the exome-wide median by more than 5%, indicative for positive selection of cancer genes (Supplementary information 2). In addition, we examined their mutational patterns in gene networks, providing important insights on gene interactions and disease drivers. We determined network associations of somatic mutations at the systems level by gene set enrichment analysis^12^. Detected somatic mutations were significantly enriched in the G_αs_ stimulatory heterotrimeric guanine nucleotide-binding protein (G_s_-protein) pathway, M14775, with a q-value below 1.0e-6. This pathway includes BRAF, RAF1, cAMP-responsive element binding proteins CREB3 (Gene ID: 10488) and CREB5 (Gene ID: 9586), and mitogen-activated protein kinase 1, MAPK1 (Gene ID: 5594). In addition, the cyclin pathway, M1529, including mutually exclusive mutations of cyclin-dependent kinase CDK4, its inhibitor CDKN2A, proline-rich protein BstNI subfamily 1 PRB1, and tumor suppressor TP53 showed statistically significant perturbation with a q-value below 1.0e-6. The assessment of the mutational pattern in SKCM patients showed strong mutual exclusivity of TMEM216 with members of the MAPK pathway (Supplementary information 3).

### Recurrent somatic splice site mutation of TMEM216

The transmembrane protein 216 (TMEM216) is required for tissue-specific ciliogenesis and may regulate ciliary membrane composition^13^,^14^. TMEM216 is the melanoma gene with the most significant synonymous somatic mutations of the TCGA SKCM dataset. TMEM216 is mutated at the highly conserved region between transmembrane helix 1 and 2 in 8 out of 276 SKCM patients (3% patient mutation frequency, p-value 6.5e-11, q-value 9.80e-8) at a single site at coding base position 138 from T to G, located at the 3′ splice site (Figure 5). The 3′ exon recognition at the acceptor splice site is critical for U2AF1 (U2 small nuclear RNA auxiliary factor 1, Gene ID: 7307) interaction^15^. The c.T138G replacement creates a mutation in the 3′ exon splice site of TMEM216. In a different TCGA dataset, the same significant, high-frequency nucleotide replacement is observed in 3 of 289 patients with lower grade glioma (LGG) (Figure 5, Supplementary table 7).

### Concerted deregulation of G-protein signaling and MAPK cascade stimulates melanogenesis

**Figure 6:**
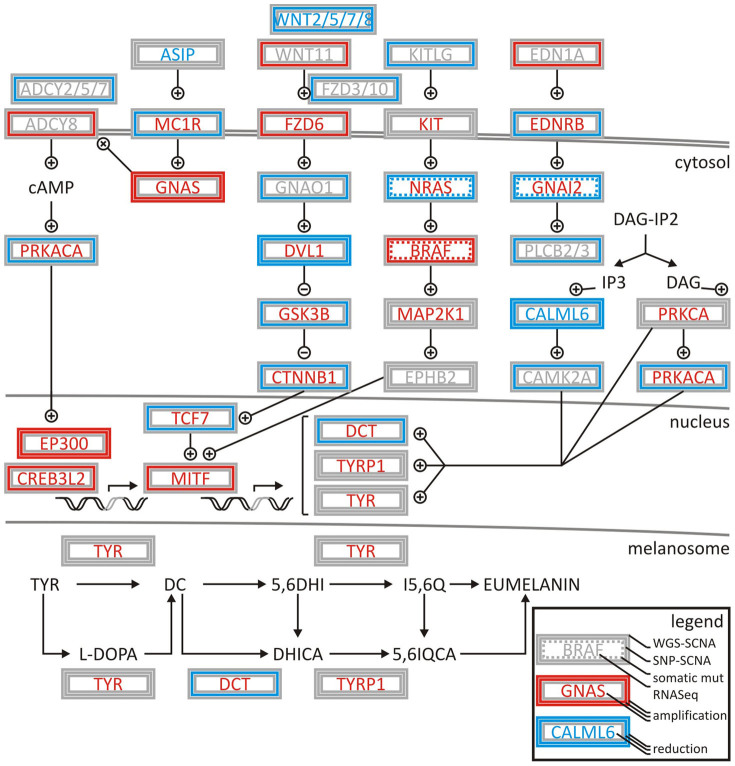
Deregulation of melanogenesis by G-protein and cyclin pathway signalling in TCGA SKCM patient samples. Coordination of signaling events is demonstrated by integrating low coverage whole genome sequencing somatic copy number alteration (WGS SCNA) data, SNP somatic copy number alteration (SNP SCNA) data, somatic mutations, and RNASeq gene expression analysis. Amplifications or activations are indicated in red, deletions or reductions in blue, non-silent mutations by dashed line. Genes are boxed by analysis type. High or low activity (gene expression) is indicted by red or blue font. Grey indicated undefined state in patient cohort. + and – symbols indicate activation and inhibition of factors in the normal pathway. The network is assembled based on gene-associations of map hsa04916 and entries of M14775 and M1529 identified by systems biology gene set enrichment analysis.

Integrated systems biology analysis including somatic copy number alterations as well as pathway enrichment of somatic mutations point towards main signaling axes in melanoma; G_s_-protein and MAPK signaling are frequently dysregulated in SKCM. The genomic observation in SKCM of strong mutual exclusivity of NRAS to BRAF is consistent with NRAS mutations activating both effector cascades BRAF/MEK/ERK and PI3K/Akt^11^,^16^. The analysis identified somatic mutations and copy number alterations affecting the G_s_-protein pathway in more than 80% of the tumors. Included were activating somatic point mutations and copy number amplification of BRAF (50%, responsive to MAPK signaling pathway activator), mutation and deletion of NRAS (31%, responsive to MAPK signaling pathway activator), as well as mutation and deletion of GNAI2 (guanine nucleotide binding protein (G protein), alpha inhibiting activity polypeptide 2, Gene ID: 2771) (2%, responsive to GTP activator). GNAI2 proteins contribute to malignant cell growth, and its inactivation can inhibit proliferation of melanoma cells and possibly that of other malignant cells both *in vitro* and *in vivo*^17^. So far statistically significant mutation of GNAI2 in melanoma has not been reported. It is a potential therapeutic target and needs further studies to assess its clinical significance. In addition, CAMP, CREB3, CREB5, MAPK1, and RAF1 are mutated in the G_s_-protein pathway in more than 10% of the tumors. Other mutations, genomic amplifications, or deletions that affect melanogenesis pathways included somatic copy number amplification in genes FZD6, GNAS, EP300, CREB3L2 and MITF. MITF is the top hit of the SCNA analysis and a critical signaling hub involved in melanocyte development, survival, and melanogenesis (Figure 6). Increased expression of MITF and its activation by phosphorylation activates the transcription of melanocyte-specific proteins TYR, TYRP1 and DCT. Other somatic copy number deletions occurred in genes MC1R, DVL1, CALML6, and DCT.

## Discussion

### Sample size of highly mutated cancer

The TCGA landmark study across many cancer types revealed that the universe of cancer mutations is much bigger than previously thought. In the case of melanoma, however, comprehensive cataloguing of low-frequency mutated genes (in ~2% patients) will require more than 5000 samples^18^. The SKCM study with about 300 samples nearly doubles the existing pool and adds value to the growing list of whole-exome sequenced melanoma^9^,^10^,^19^,^20^,^21^,^22^.

Most genes are mutated at intermediate frequencies, creating a challenge for melanoma samples with high mutation rate of 12–18 mutations per Mbp (TCGA SKCM this study: 18/Mbp, Broad Institute's melanoma study: 12.9/Mbp^18^). A filter-based strategy helped to control for passenger mutational load (Table 1, Figure 2). Using this strategy, we were able to characterize in-depth predominant melanoma drivers—BRAF, NRAS, PTEN, TP53, CDKN2A—and also validate recently identified melanoma genes, RAC1(p.P29S)^9^,^10^ and STK19(p.D89N)^9^. Among 10 newly identified melanoma genes, SERPINB3 is a suicide-substrate protease inhibitor, which balances cell survival and apoptosis, and is shown to be up-regulated in breast, liver, cervical, lung, and other cancers^23^. The high level of genomic instability is evidenced by significantly increased frequencies of SCNA (Figure 1). We detected concurrent arm-level alteration of both arms of chromosomes 6, 20, 9, 10, 11 indicative of aneuploidy. In addition to aneuploidy or focal SCNA events, significant enrichment of inactivating TP53 may contribute to other classes of structural variations, like breakage or fusions. While SCNAs alone cannot infer on history or heterogeneity of the tumor, future analysis on clonality status may provide additional weight to identified drivers.

### New cancer drivers

TMEM216 stands out among the newly discovered genes in melanoma as a low-frequency, highly statistically significant, recurrent splice site mutation. The mutation c.T138G in the splice site joining exons 2 and 3 disrupts the recognition motif of splicing auxiliary factor U2AF1^15^,^24^. The importance of the splice site mutation is further enhanced by the nature of the non-UV induced nucleotide change, strong mutual exclusivity with known oncogenes, and recurrence in non-melanoma cancer tissues. Germline mutations of TMEM216 (or MKS2) cause human ciliopathies like Meckel–Gruber syndrome (MKS) or Joubert syndrome (JBTS)^13^ and revealed localization of TMEM216 to Golgi vesicles necessary for ciliary assembly^25^. Cilia are important organelles of cells and are involved in numerous activities such as cell signaling and processing developmental signals. Inactive TMEM216 hyper-activates RHOA signaling and increases phosphorylation of the planar cell polarity pathway of non-canonical Wnt signaling protein Dishevelled, DVL1^14^,^25^,^26^. In the context of melanoma, somatic splice-site mutation of TMEM216 suggests potential tumor suppressor function and sets the RHOA/GNA12 pathway apart from the G_s_-protein/MAPK pathway by mutual exclusivity of TMEM216 towards known oncogenes NRAS and RAC1 (Supplementary information 3).

### Pan-cancer

Somatic missense mutation of BRAF has been identified in roughly half of all malignant melanoma cases and at much lower frequency in all other cancers^2^,^9^,^10^. Today, whole-genome and -exome sequencing allows deep insight into molecular carcinogenesis of melanoma. The TCGA SKCM study adds to existing genome studies where BRAF(p.V600E) has been identified in 52.9% of the samples of the melanoma study of the Broad Institute^9^, 64.0% in the Harvard study^22^, 45.9% in the Yale study^10^, as well as in 20.8% of all NCI60 cell lines^27^.

Previously reported mutations rarely fell outside the kinase domain, with the coding substitution of p.V600E accounting for more than 80% of reported cases^2^. Our detailed and targeted sampling of the mutational landscape of BRAF in TCGA melanoma as well as in all other tissues of TCGA by next-generation sequencing brought three main insights forward: a) BRAF is significantly increased at a copy-number level and constitutively activated by somatic mutations; b) The whole-exome data showed unprecedented diverse mutational events within BRAF (Figure 3) aside from p.V600E preserving mutual exclusivity to activating NRAS mutations; c) Other cancers have similar or even stronger BRAF signatures than melanoma.

These observations manifest BRAF as a *bona fide* pan-cancer driver and have consequences for BRAF as an anti-cancer target and diagnostic marker. Lessons already learned from molecular studies of the BRAF pathway are relevant to almost all cancer tissues, which showed somatic missense mutations. The diverse mutational landscape challenges medicinal chemistry efforts to develop new compounds recognizing the different molecular conformations of mutated activator and ATP binding loop aside from established p.V600E binders. A single SNP test for c.1799T>A is not sufficient to capture the majority of mutational events of BRAF in its RBD or in the ATP binding site of its PK domain. Datasets like TCGA lung squamous cell carcinoma (LUSC) show mutational incidents of BRAF in more than 5% of the patients but not a single substitution of c.1799T>A. On a positive note, the diagnostic value of c.1799T>A for BRAF diagnostics is widened to many other cancers besides SKCM that homogeneously show single p.V600E substitutions like THCA, COAD, GBM, KIRP, READ, and LGG.

## Conclusion

The systems biology analysis of melanoma showed an unprecedented richness and depth of statistically significant and novel melanoma genes (Figure 2). By combining established tools in genomics, we were able to create a multi-step filter that accounts for enrichment in functional hot spots as well as the elevated and nucleotide-specific basal rate due to UV damage (Supplementary information 2). The integrated analysis of somatic mutations and structural genomic alterations in melanogenesis showed coordination of proliferative signaling events in mutually exclusive settings in melanoma (Figure 6, Supplementary information 3). If driver mutations are observed in other cancer tissues as well, lessons learned on regulation of signaling cascades and drug resistance of cancer targets might be directly translatable. Thyroid cancer showed an enrichment of the somatic melanoma driver mutation BRAF(p.V600E) that surpassed profiles of BRAF of any other tissue (Figure 3–4), while preserving mutually exclusive setting to RAS mutations (Supplementary table 6). For patients with activated BRAF pathways in their thyroid tissues, knowledge on molecular medicine of the BRAF cascade in melanoma becomes highly valuable. The systems biology integration of genomic alterations in melanoma provides a glimpse into how a spectrum of genomic aberrations contributes to melanoma genesis and progression (Figure 6). Identification of such genomic aberrations in melanoma patients contributes to new treatment regimens based on molecular understanding of driver events that govern this malignancy.

## Methods

### The Cancer Genome Altas project

The study was carried out as part of IRB approved study dbGap ID 5094 “Somatic mutations in melanoma”. The results shown are in whole based upon data generated by the TCGA Research Network http://cancergenome.nih.gov. Restricted access whole-genome sequences and whole-exome sequences were obtained from the TCGA data portal.

### Somatic copy number alterations

The tool GISTIC 2.0.21^7^,^8^ was used to identify genomic regions that are significantly gained or lost across a set of paired normal and tumors samples of TCGA SKCM data set (for abbreviations see glossary). We executed GISTIC 2.0.21 on Illumina HiSeq data recorded with a low coverage whole-genome sequencing protocol, MD Anderson Cancer Center, TX, as well as on Agilent SNP 6.0 gene expression microarrays G4502A_07_01, UNC Chapel Hill, NC. GISTIC 2.0.21 distinguishes arm-level events from focal events at a broad length cutoff of 0.7. Events whose length was greater or less than 50% of the chromosome arm on which they resided were called arm-level or focal events, respectively, and these groups of events were analyzed separately. The data was concordant to segmented level 3 data publically available at the TCGA data portal. Since GISTIC 2.0.21 uses ratios of segmented tumor copy number data relative to normal samples as input, segmented level 3 data aligned to HG19 served as input for analysis runs. For significant loci and genes a cutoff q-value of 0.01 was applied, and concordance determined by overlaying whole-genome sequencing and SNP data. All experiments on SCNAs were carried out at a confidence level of 0.99 according to established standards by the TCGA Research Network and compared to benchmarks established by the Broad Institute TCGA Genome Characterization Center http://www.broadinstitute.org/collaboration/gcc and Broad Institute GDAC Firehose pipeline https://confluence.broadinstitute.org/display/GDAC.

### Multi-step filter for somatic mutations

We applied a multi-step filter to identify somatic mutations (Table 1). Critical components of the computational filters were i) TCGA data portal for cohort selection and CGHub for access of raw data; ii) MuTect 1.1.4 at default settings for preprocessing, alignment of reads in the tumor and normal sequencing data, and mutation calling^28^; iii) MutSig 2.0, an algorithm for identification of mutation significance, for assessing the clustering of mutations in hotspots as well as conservation of the sites^29^; and iv) InVEx 1.0.1, a permutation based Intron vs Exon algorithm for ascertaining positive selection of somatic mutation above the background level considering heterogeneity on a per-patient and per-gene level^9^. Functional impact was assessed by v) UV biased nucleotide signature^10^; vi) structural modeling using SWISS-MODEL http://swissmodel.expasy.org/ and TMpred http://www.ch.embnet.org; vii) GSEA 2.1.0 for gene set enrichment analysis^12^ and MEMo 1.1 for mutual exclusivity modules analysis^11^; as well as viii) recurrence in other TCGA tissues.

i) Whole-exome sequencing files wes.bam in compressed binary version of sequence alignment map SAM (BAM) format for 276 TCGA patients (barcodes provided in Supplementary table 1) collected on Illumina HiSeq platforms recorded at the MIT Broad Institute, MA or Biospecimen Core Resource collected by TCGA consortium were downloaded from CGHub, Cancer Genomics Hub Browser, hosted at the University of California, Santa Cruz. Each sample from a tumor metastatic cancer (TM) was matched with a normal blood derived sample (NB). ii) For the MuTect 1.1.4 analysis^28^ GrCh37 (Genome Reference Consortium Human Reference 37, Broad Institute variant of human genome assembly 19 (HG19)), SNP database (dbSNP) build 132.vcf, and catalogue of somatic mutations in cancer (COSMIC_54.vcf) library were referenced. dbSNP build 132.vcf is a database referenced to GrCh37 of known human germline variations derived from the 1000 genomes project. COSMIC_54.vcf is a database referenced to GrCh37 of somatically-acquired mutations found in human cancer. The call_stats.txt files containing the list of all the mutations per patient and coverage.wig in wiggle file format were generated for every matched sample as an output of MuTect 1.1.4. The mutation call_stats.txt file was queried in bash prompt to retain all the statically significant KEEP mutations under standard settings i.e. ensuring coverage of 80% power for a 0.3 allelic fraction mutation. iii) MutSig 2.0 executed on whole-exome Illumina HiSeq DNA sequencing data accesses three main sources of evidence in the data to estimate the amount of positive selection a gene underwent during tumorigenesis: 1. Abundance of mutations relative to the background mutation rate, 2. Clustering of mutations in hotspots within the gene, and 3. Conservation of the mutated positions. MutSig 2.0 was the method of choice for the SKCM study, because it has augmented sophisticated procedures for treating the heterogeneity in per-gene, per-patient, and per-context background mutation rate. Evidence of conservation and clustering are examined by a separate part of MutSig 2.0 that performs many permutations. Mutations were inferred from raw binary alignment wes.bam files and compared to benchmarks at the cancer genome analysis multi-pipeline Firehose, which performs analyses including quality control, local realignment, single nucleotide variations identification, insertion and deletion identification, as well as inter-chromosomal and large intra-chromosomal structural rearrangement detection and mutation rate calculation. From 220,031 exonic mutations, Mutsig 2.0 produces a list of significantly mutated genes, covariates.txt. iv) The permutation algorithm InVEx 1.0.1, Intron vs Exon, was employed to efficiently model the somatic mutation rate among genes to identify the genes that most frequently harbor non-silent mutations^9^. InVEx 1.0.1 permutes coding, untranslated, and intronic mutations per nucleotide for each gene in all the patients to generate a list of genes that have the most functional impact. The polymorphism phenotyping version 2 (PPH2) library, human genome HG19, nucleotide_classes_HG19.txt, genePeptideFile_HG19, and COSMIC_54.vcf library are used as references. The coverage.wig and covariates.txt files were used as an input for InVEx 1.0.1. The QQ-plot qq.png of functional mutation burden and synonymous mutation burden significant_mutation_burden.txt were produced as an outcome of InVEx 1.0.1.

The list of enriched genes significant_mutation_burden.txt produced by filtering steps i)-iv) is analyzed for functional impact. v) Deviation from the exome-wide median of the signature of nucleotide replacement can be indicative for positive selection of cancer genes. In particular, increased transitions from cytidine to thymidine (C->T) characterize an ultraviolet-induced mutational signature (Supplementary information 2). The mutation annotation file patient.maf entries for target genes were sorted by transition type and filtered for UVA (C>A) or UVB (C>T) mediated transitions. vi) Mutations were plotted on existing experimental or modeled structures using SWISS-MODEL. In the case of transmembrane proteins, the transmembrane topology was assessed using TMpred. vii) Impact of pathways was assessed by GSEA 2.1.0 and MEMo 1.1 using scna.txt matrix file, amplified and deleted genes amp_del_gene.txt of GISTIC 2.0.21, coverage.wig file from MuTect 1.1.4, covariates.txt from Mutsig 2.0, while referencing the tcga_patient_list.txt, with a q-value threshold of 0.10 and 10 alterations. Absolute expression levels of RNASeq data from 302 patients were assessed using 5^th^ or 95^th^ percentile thresholds for lowly or highly expressed genes, respectively. The SKCM dataset at the TCGA datahub only contained a somatic reference file for 1 of 302 patients with metastatic melanoma at the point of the analysis. viii) For driver genes of covariates.txt in SKCM with high mutational burden in i)-iv) as well as functional impact in v)-vii), all patient.maf files in TCGA were searched for recurrence in other cancer tissues. The results are sorted covariate.maf tables for each cancer driver (Supplementary tables 9, 10).

## Author Contributions

F.V.F. designed the study and wrote the main article text. J.G., R.G., F.V.F. performed the data analysis of the SKCM TCGA dataset, prepared methods and supplementary information, and reviewed the final manuscript.

## Supplementary Material

Supplementary Information Supplementary information

Supplementary Information Supplementary tables

## Figures and Tables

**Table 1:** Multi-step filter selects cancer drivers in genomics datasets with high mutational burden

Step	Logic	Program	Input	Output	Reference files
i	Cohort selection	TCGA Portal, CGHub, Gene Torrent	tcga_patient_list.txt	wes.bam	
ii	Mutation call	MuTect 1.1.4^28^	wes.bam	coverage.wig, call_stats.txt	HG19, COSMIC_54.vcf, dbsnp_132.vcf
iii	Recurrence, evolutionary conservation	MutSig 2.0^29^	patient.maf, coverage.wig	covariates.txt	HG19/.maf
iv	Correction for background mutation rate	InVEx 1.0.1^9^	coverage.wig, covariates.txt	significant_mutation_burden.txt, qq.png	HG19/.maf, PPH2, nucleotide_classes_HG19.txt, COSMIC, genePeptideFile_HG19
v	Mutation Signature, UV induced damage	Text editor	patient.maf	sorted_transitions.maf	nucleotide_classes_HG19.txt, uv_transitions.txt^10^
vi	Structure-activity-relationship	SWISS-MODEL 8.05, TMpred, 25.0	structure.pdb	model.pdb, tm_model.txt	
vii	Pathway enrichment, Mutual exclusivity	GSEA 2.1.0^12^, MEMo 1.1^11^	tcga_patient_list.txt, scna.txt, amp_del_gene.txt, covariates.txt, coverage.wig	modules.txt	
viii	Recurrence within PAN-Cancer TCGA	TCGA	covariate_target.txt, patient.maf	covariate.maf	

Somatic mutations of driver genes are called after i) cohort selection, ii) mapping of human genome and patient specific somatic references, iii) assessment of recurrence, evolutionary conversation, and iv) basal mutation rate based on frequency of mutations of introns vs exons. This first set of filters i)-iv) is necessary and sufficient to identify statistically significant enriched somatic mutations of driver genes in any dataset with high mutational burden. In a genome-wide sequencing experiment with a goal to find cancer drivers, an additional level of filters v)-viii) is advantageous. Relevance of mutations is assessed by v) nucleotide signature, vi) structure activity relationship, vii) pathway enrichment and mutual exclusivity to known cancer drivers, as well as viii) recurrence in other cancer tissues.

## References

[b1] Meyerson M., Gabriel S. & Getz G. Advances in understanding cancer genomes through second-generation sequencing. Nat Rev Genet 11, 685–696 (2010). 20847746 10.1038/nrg2841

[b2] Davies H. *et al.* Mutations of the BRAF gene in human cancer. Nature 417, 949–954 (2002). 12068308 10.1038/nature00766

[b3] Pollock P. M. *et al.* High frequency of BRAF mutations in nevi. Nat Genet 33, 19–20 (2003). 12447372 10.1038/ng1054

[b4] Chapman P. B. *et al.* Improved survival with vemurafenib in melanoma with BRAF V600E mutation. N Engl J Med 364, 2507–2516 (2011). 21639808 10.1056/NEJMoa1103782 PMC3549296

[b5] Flaherty K. T. *et al.* Improved survival with MEK inhibition in BRAF-mutated melanoma. N Engl J Med 367, 107–114 (2012). 22663011 10.1056/NEJMoa1203421

[b6] Sun C. *et al.* Reversible and adaptive resistance to BRAF(V600E) inhibition in melanoma. Nature 508, 118–122 (2014). 24670642 10.1038/nature13121

[b7] Mermel C. H. *et al.* GISTIC2.0 facilitates sensitive and confident localization of the targets of focal somatic copy-number alteration in human cancers. Genome Biol 12, R41 (2011). 21527027 10.1186/gb-2011-12-4-r41 PMC3218867

[b8] Beroukhim R. *et al.* The landscape of somatic copy-number alteration across human cancers. Nature 463, 899–905 (2010). 20164920 10.1038/nature08822 PMC2826709

[b9] Hodis E. *et al.* A landscape of driver mutations in melanoma. Cell 150, 251–263 (2012). 22817889 10.1016/j.cell.2012.06.024 PMC3600117

[b10] Krauthammer M. *et al.* Exome sequencing identifies recurrent somatic RAC1 mutations in melanoma. Nat Genet 44, 1006–1014 (2012). 22842228 10.1038/ng.2359 PMC3432702

[b11] Raphael B. J., Dobson J. R., Oesper L. & Vandin F. Identifying driver mutations in sequenced cancer genomes: computational approaches to enable precision medicine. Genome Med 6, 5 (2014). 24479672 10.1186/gm524 PMC3978567

[b12] Subramanian A. *et al.* Gene set enrichment analysis: a knowledge-based approach for interpreting genome-wide expression profiles. Proc Natl Acad Sci U S A 102, 15545–15550 (2005). 16199517 10.1073/pnas.0506580102 PMC1239896

[b13] Hildebrandt F., Benzing T. & Katsanis N. Ciliopathies. N Engl J Med 364, 1533–1543 (2011). 21506742 10.1056/NEJMra1010172 PMC3640822

[b14] Garcia-Gonzalo F. R. *et al.* A transition zone complex regulates mammalian ciliogenesis and ciliary membrane composition. Nat Genet 43, 776–784 (2011). 21725307 10.1038/ng.891 PMC3145011

[b15] Wu S., Romfo C. M., Nilsen T. W. & Green M. R. Functional recognition of the 3' splice site AG by the splicing factor U2AF35. Nature 402, 832–835 (1999). 10617206 10.1038/45590

[b16] Marzese D. M. *et al.* DNA methylation and gene deletion analysis of brain metastases in melanoma patients identifies mutually exclusive molecular alterations. Neuro Oncol. 16, 1499–509 (2014). 24968695 10.1093/neuonc/nou107 PMC4201072

[b17] Hermouet S., Aznavoorian S. & Spiegel A. M. In vitro and in vivo growth inhibition of murine melanoma K-1735 cell by a dominant negative mutant alpha subunit of the Gi2 protein. Cell Signal 8, 159–166 (1996). 8736698 10.1016/0898-6568(95)02049-7

[b18] Lawrence M. S. *et al.* Discovery and saturation analysis of cancer genes across 21 tumour types. Nature 505, 495–501 (2014). 24390350 10.1038/nature12912 PMC4048962

[b19] Nikolaev S. I. *et al.* Exome sequencing identifies recurrent somatic MAP2K1 and MAP2K2 mutations in melanoma. Nat Genet 44, 133–139 (2012). 22197931 10.1038/ng.1026

[b20] Pleasance E. D. *et al.* A comprehensive catalogue of somatic mutations from a human cancer genome. Nature 463, 191–196 (2010). 20016485 10.1038/nature08658 PMC3145108

[b21] Wei X. *et al.* Exome sequencing identifies GRIN2A as frequently mutated in melanoma. Nat Genet 43, 442–446 (2011). 21499247 10.1038/ng.810 PMC3161250

[b22] Berger M. F. *et al.* Melanoma genome sequencing reveals frequent PREX2 mutations. Nature 485, 502–506 (2012). 22622578 10.1038/nature11071 PMC3367798

[b23] Catanzaro J. M. *et al.* Elevated expression of squamous cell carcinoma antigen (SCCA) is associated with human breast carcinoma. PLoS One 6, e19096 (2011). 21526154 10.1371/journal.pone.0019096 PMC3079753

[b24] Brooks A. N. *et al.* A pan-cancer analysis of transcriptome changes associated with somatic mutations in U2AF1 reveals commonly altered splicing events. PLoS One 9, e87361 (2014). 24498085 10.1371/journal.pone.0087361 PMC3909098

[b25] Lee J. H. *et al.* Evolutionarily assembled cis-regulatory module at a human ciliopathy locus. Science 335, 966–969 (2012). 22282472 10.1126/science.1213506 PMC3671610

[b26] Valente E. M. *et al.* Mutations in TMEM216 perturb ciliogenesis and cause Joubert, Meckel and related syndromes. Nat Genet 42, 619–625 (2010). 20512146 10.1038/ng.594 PMC2894012

[b27] Abaan O. D. *et al.* The exomes of the NCI-60 panel: a genomic resource for cancer biology and systems pharmacology. Cancer Res 73, 4372–4382 (2013). 23856246 10.1158/0008-5472.CAN-12-3342 PMC4893961

[b28] Cibulskis K. *et al.* Sensitive detection of somatic point mutations in impure and heterogeneous cancer samples. Nat Biotechnol 31, 213–219 (2013). 23396013 10.1038/nbt.2514 PMC3833702

[b29] Lawrence M. S. *et al.* Mutational heterogeneity in cancer and the search for new cancer-associated genes. Nature 499, 214–218 (2013). 23770567 10.1038/nature12213 PMC3919509

