# Supplementary material for: Cancer systems biology of TCGA SKCM: Efficient detection of genomic drivers in melanoma
Source: Sci Rep. 2015 Jan 20;5:7857. doi: 10.1038/srep07857 (PMC4298731; doi:10.1038/srep07857)
Supplement: Supplementary Information [file srep07857-s1.pdf]

Cancer systems biology of TCGA SKCM: Efficient detection of potential genomic drivers in melanoma

Systems Biology and Cancer Metabolism, Program for Quantitative Systems Biology, University of California Merced, Merced, CA 95343, USA

Jian Guan, Rohit Gupta, Fabian V. Filipp

e-mail: filipp@ucmerced.edu

Supplementary information—*Table of contents*

Supplementary information 1—*Hotspot somatic mutation of the leucine zipper of LUZP2*

Supplementary information 2—*Signature of UV mutagenesis across driver mutations in melanoma*

Supplementary information 3—*Mutual exclusivity of driver mutations in TMEM216 in melanoma*

Supplementary References

Supplementary Tables

Supplementary Table 1—*Patient barcodes of SKCM TCGA project*

Supplementary Table 2—*Somatic copy number alterations SNP array amplification*

Supplementary Table 3—*Somatic copy number alterations LowPass amplification*

Supplementary Table 4—*Somatic copy number alterations SNP array deletion*

Supplementary Table 5—*Somatic copy number alterations LowPass deletion*

Supplementary Table 6—*Somatic mutations of BRAF in TCGA*

Supplementary Table 7—*Somatic mutations of TMEM216 in TCGA*

Supplementary Table 8—*Somatic mutations of LUZP2 in TCGA*

Supplementary Table 9—*Functional somatic mutations in TCGA SKCM*

Supplementary Table 10—*Synonymous somatic mutations in TCGA SKCM*

Supplementary information 1—*Hotspot somatic mutation of the leucine zipper of LUZP2*

The leucine zipper protein 2 (LUZP2) has been reported to be deleted in some patients with WAGR syndrome, a genetic disorder with predisposition towards *W*ilms tumor, *A*niridia, *G*enitourinary anomalies, and mental *R*etardation [1, 2]. The SKCM dataset contains in total 34 non-silent somatic mutations of LUZP2 affecting 25 unique residues (9% patient mutation frequency, p-value 2.29e-09, q-value 2.75e-06). The leucine zipper region of LUZP2 harbors the recurring mutation p.R165C. There are 10 additional mutation sites close to the leucine zipper region between residues 140 and 213. Mutation of the recurring site R165 is observed as well in COAD, where p.R165H is mutated. The mutation of LUZP2 is mutually exclusive to other cancer genes like NRAS, TMEM216, OXA1L, and LCE1B. The replacement p.R43C is a recurrent mutation found in SKCM and COAD. For SKCM as well as in the context of the Pan-Cancer study the mutations are about two-fold enriched above random distribution of the sequence in the leucine-zipper domain of LUZP2.

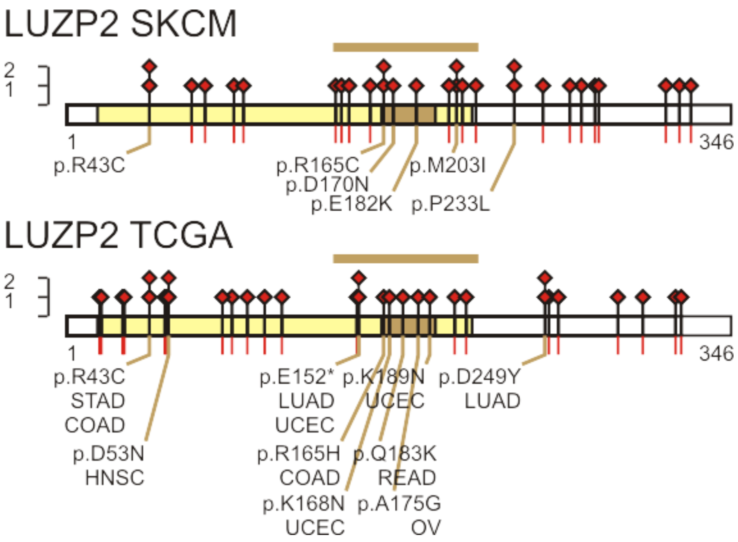

Supplementary information 1 Figure: LUZP2 is hyper-mutated in SKCM with recurring somatic mutations in the leucine zipper region

Somatic mutations are indicated in red on the protein sequence of LUZP2, NCBI Gene ID 338645, for SKCM and other TCGA tissues. Leucine zipper domain and coiled-coil regions are indicated in gold and yellow, respectively, on the sequence according to Uniprot entry Q86TE4. All recurrent mutations as well as mutations in the proximity of the leucine zipper are marked in gold below the sequence. A golden bar indicates the hyper-mutated region in the proximity of the leucine zipper.

Supplementary information 2—*Signature of UV mutagenesis across driver mutations in melanoma*

Deviation from the exome-wide median of the signature of nucleotide replacement can be indicative for positive selection of cancer genes. In particular, increased transitions from cytidine to thymidine (C->T) characterize an ultraviolet-induced mutational signature [3, 4, 5, 6]. UV-induced DNA damage in conjunction with DNA mismatch repair are a common cause of somatic mutations in melanoma, raising the frequency of UVA and UVB associated mutations about four-fold to 83% in comparison to other TCGA cancers [3]. We quantified the distribution of mutations attributable to UVA radiation causing G>T mutations and UVB causing C>T mutations. TMEM216, BRAF, PTEN, NRAS, TP53, LUZP2, RAC1, and CDKN2A show UV-related nucleotide replacement below the sample median indicating proliferative advantage of the somatic mutation. Further PTEN, NRAS, CDKN2A, SKT19, and KNSTRN show elevated UVA or UVB signature, connecting these genes with the exposure to carcinogenic exposure of ultraviolet light.

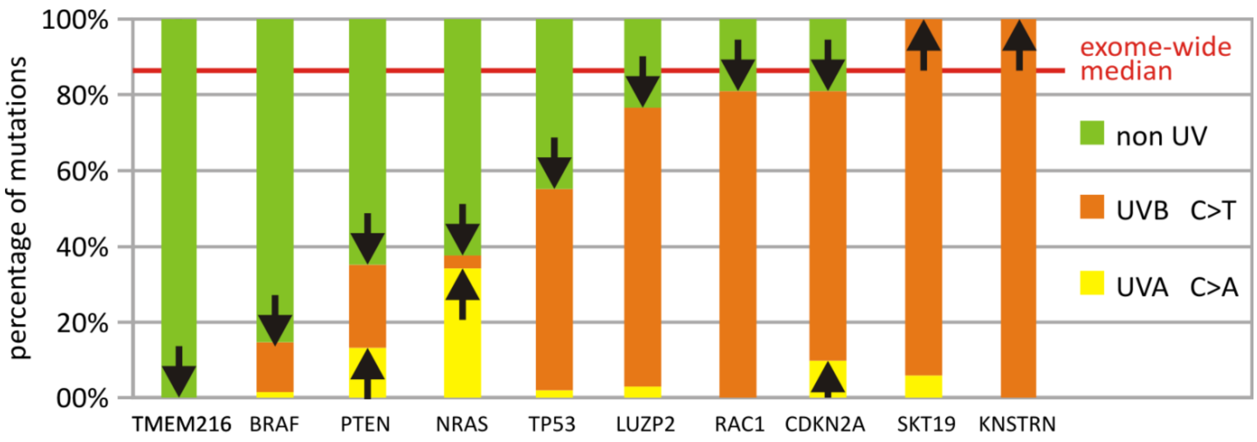

Supplementary information 2 Figure: Signature of UV mutagenesis in melanoma drivers

Percentage of driver mutations caused by UVA (C>A) or UVB (C>T) are plotted vs the exome-wide sample average percentage. Genes with non UV mutations deviating from exome-wide sample average are marked with an arrow.

Supplementary information 3—*Mutual exclusivity of driver mutations in TMEM216 in melanoma*

The TCGA SKCM study confirmed a dominance of somatic mutations in BRAF of 50% of patients. In 29% of the patients NRAS is mutated in a mutually exclusive setting to BRAF. The NRAS gene was mutated in 86 cases (85 missense, 1 splice-site mutation) which is contributed to 31% all melanoma cases. The genomic observation of strong mutual exclusivity is consistent with NRAS mutations activating both effector cascades BRAF-MEK-ERK and PI3K-Akt [7, 8]. Examination of pattern information of somatic mutations in gene networks provides important information on gene interactions and disease drivers. The assessment of the mutational pattern in SKCM patients showed strong mutual exclusivity of TMEM216 with other significantly mutated genes.

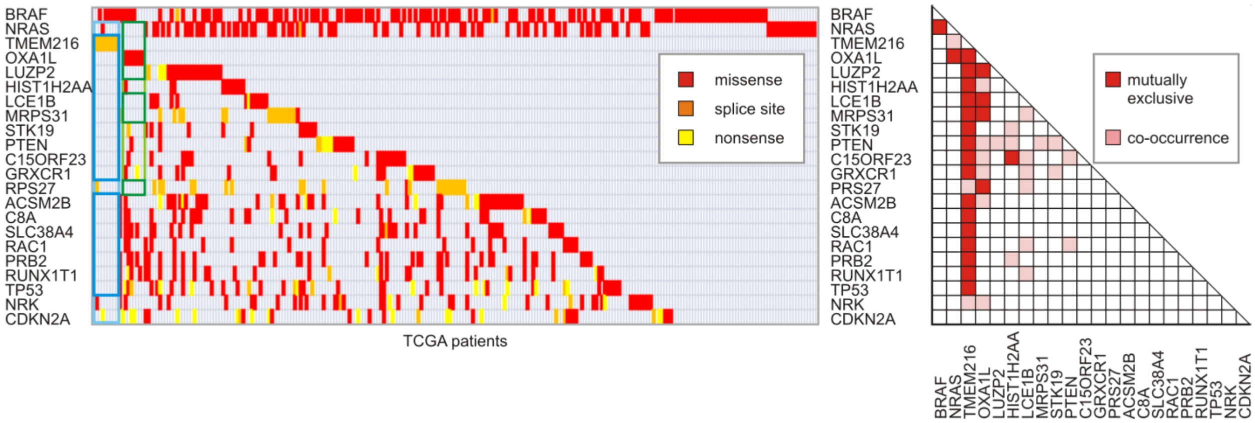

Supplementary information 3 Figure: Mutual exclusivity of driver mutations in TMEM216 in melanoma

Combinations of mutations are evaluated by focusing on pattern of co-occurrence or mutual exclusivity. Mutation matrix shows functional mutations of frequently mutated genes for 276 patients of SKCM dataset. Missense mutations shown in red, splice site mutations in orange, and nonsense mutations in yellow. Lack of co-occurrence is indicated by a dark blue boxes around recurring TMEM216 mutations and dark green boxes for LUZP2. Pair-wise matrix shows existence of mutual exclusive interaction. Red box indicates mutual exclusive mutations in different patients. Light red indicates co-occurrence of only one patient with mutation of both genes of interest. White box indicates co-occurrence of mutations.

Supplementary References

1. Wu M, Michaud EJ, Johnson DK. Cloning, functional study and comparative mapping of Luzp2 to mouse chromosome 7 and human chromosome 11p13-11p14. *Mamm Genome* **14**, 323-334 (2003).

2. Stefan M*, et al.* Genetic mapping of putative Chrna7 and Luzp2 neuronal transcriptional enhancers due to impact of a transgene-insertion and 6.8 Mb deletion in a mouse model of Prader-Willi and Angelman syndromes. *BMC Genomics* **6**, 157 (2005).

3. Lawrence MS*, et al.* Mutational heterogeneity in cancer and the search for new cancer-associated genes. *Nature* **499**, 214-218 (2013).

4. Hodis E*, et al.* A landscape of driver mutations in melanoma. *Cell* **150**, 251-263 (2012).

5. Berger MF*, et al.* Melanoma genome sequencing reveals frequent PREX2 mutations. *Nature* **485**, 502-506 (2012).

6. Pleasance ED*, et al.* A comprehensive catalogue of somatic mutations from a human cancer genome. *Nature* **463**, 191-196 (2010).

7. Raphael BJ, Dobson JR, Oesper L, Vandin F. Identifying driver mutations in sequenced cancer genomes: computational approaches to enable precision medicine. *Genome Med* **6**, 5 (2014).

8. Marzese DM*, et al.* DNA methylation and gene deletion analysis of brain metastases in melanoma patients identifies mutually exclusive molecular alterations. *Neuro Oncol*, (2014).
